# TSC-22 up-regulates collagen *3a1* gene expression in the rat heart

**DOI:** 10.1186/s12872-015-0121-2

**Published:** 2015-10-13

**Authors:** Annina Kelloniemi, Jani Aro, Juha Näpänkangas, Elina Koivisto, Erja Mustonen, Heikki Ruskoaho, Jaana Rysä

**Affiliations:** Research Unit of Biomedicine (Pharmacology & Toxicology), University of Oulu, Oulu, Finland; Department of Pathology, Institute of Diagnostics, Oulu University Hospital, University of Oulu, Oulu, Finland; Division of Pharmacology and Pharmacotherapy, Faculty of Pharmacy, University of Helsinki, Helsinki, Finland; School of Pharmacy, Faculty of Health Sciences, University of Eastern Finland, Kuopio, Finland

**Keywords:** Cardiac hypertrophy, Heart failure, Pressure overload, Myocardial infarction

## Abstract

**Background:**

The transforming growth factor (TGF)-β is one of the key mediators in cardiac remodelling occurring after myocardial infarction (MI) and in hypertensive heart disease. The TGF-β-stimulated clone 22 (TSC-22) is a leucine zipper protein expressed in many tissues and possessing various transcription-modulating activities. However, its function in the heart remains unknown.

**Methods:**

The aim of the present study was to characterize cardiac TSC-22 expression *in vivo* in cardiac remodelling and in myocytes *in vitro*. In addition*,* we used TSC-22 gene transfer in order to examine the effects of TSC-22 on cardiac gene expression and function.

**Results:**

We found that TSC-22 is rapidly up-regulated by multiple hypertrophic stimuli, and in post-MI remodelling both TSC-22 mRNA and protein levels were up-regulated (4.1-fold, *P* <0.001 and 3.0-fold, *P* <0.05, respectively) already on day 1. We observed that both losartan and metoprolol treatments reduced left ventricular TSC-22 gene expression. Finally, TSC-22 overexpression by local intramyocardial adenovirus-mediated gene delivery showed that TSC-22 appears to have a role in regulating collagen *type IIIα1* gene expression in the heart.

**Conclusions:**

These results demonstrate that TSC-22 expression is induced in response to cardiac overload. Moreover, our data suggests that, by regulating collagen expression in the heart *in vivo*, TSC-22 could be a potential target for fibrosis-preventing therapies.

## Background

Various stimuli such as increased mechanical stress, neurohumoral activation or myocardial injury initiate pathological alterations in the heart, referred to as cardiac remodelling [1]. Common features of pathological cardiac remodelling include alterations in cardiomyocyte size, modulation of the extracellular matrix (ECM), inflammation, and microvascular perturbations [1]. Cardiac remodelling leads to further deterioration of cardiac function, and eventually to heart failure, one of the leading causes of mortality and morbidity worldwide [1, 2].

At molecular level, cardiac remodelling is the result of altered gene expression that is regulated by the activation of numerous transcription factors including GATA-4, myocyte enhancer factor-2, Nkx2.5, serum response factor and SMADs [3]. Transcription factors are, in turn, regulated by several signalling pathways such as mitogen-activated kinases (MAPKs) p38, extracellular signal-regulated kinase (ERK) and c-Jun N-terminal kinase (JNK) [4, 5]. One of the key mediators involved in the pathological remodelling in cardiac hypertrophy and after myocardial infarction is the transforming growth factor beta (TGF-beta) [6], which regulates cardiomyocyte hypertrophy, fibroblast proliferation, inflammatory response and extracellular matrix metabolism through direct and indirect mechanisms [7, 8]. TGF-β also regulates several cardiac transcription factors including Smad 4, Smad 6/7 and cAMP response element-binding protein (CREB1) [6, 9]. Although it has been studied extensively, the role of transcriptional regulation of pathological cardiac remodelling is not yet fully understood.

TSC-22 (TGF-β stimulated clone 22) is a member of leucine zipper proteins, first isolated as TGF-β responsive gene form mouse osteoblastic cells [10]. Although the DNA binding surface of TSC-22 differs from that of the other basic leucine zipper proteins, it can homodimerize and heterodimerize with other transcription factors such as SMADs [11–14], and it has transcriptional repression activity [11]. Several physiological roles for TSC-22 have been identified: TSC-22 is thought to be a tumour suppressor in several cancer cell lines and tumour tissue [15–21], regulating developmental processes in *Xenopus laevis* during gastrulation and in oogenesis of *Drosophila melanogaster* [14, 22], apoptosis [17, 21], and systemic cholesterol metabolism [23] as well as promoting cardiac myofibroblast differentiation and fibrosis [13]. TSC-22 is up-regulated by many stimuli, including different cytokines, fibroblast growth factor 2 (FGF2), and epidermal growth factor (EGF) [24]. In addition, elevated TSC-22 mRNA-levels have been reported in an experimental model of essential hypertension, in spontaneously hypertensive rats (SHRs) [25], and after experimental myocardial infarction in rats [26]. Thus, TSC-22 may have an important role in controlling the transcriptional response of cardiac remodelling.

Here we assessed TSC-22 expression levels in the heart and used adenovirus-mediated gene delivery in the normal rat heart in order to investigate the effects of TSC-22 on cardiac gene expression and function. In addition, we analysed the effect of TSC-22 overexpression on hypertrophic gene response in cultured neonatal rat ventricular myocytes (NRVMs). We demonstrated that multiple hypertrophic stimuli and post-MI remodelling regulate TSC-22 expression in the heart. Our data shows that TSC-22 has a role in regulating collagen *Col3a1* gene expression in the heart *in vivo*, suggesting it may be involved in the development of cardiac ECM remodelling.

## Methods

### Expression and purification of TSC-22 for antibody production

For TSC-22 (also known as TSC-22D1) protein production, the cDNA coding for rat TSC-22 (GeneBank accession number L25785) was subcloned into the *Nde*I-*Bam*HI site of pET-15b expression vector (Novagen/Merck, Darmstadt, Germany) containing an amino terminal (His)_6_-tag. The construct was transformed into the *E. coli* BL21(DE3) cells and grown in LB-medium in the presence of ampicillin (100 μg/ml) at +37 °C. Protein expression was induced by 1 mM isopropyl b-D-thiogalactoside (IPTG) for 3 h, after which the cells were harvested, suspended in a lysis buffer (100 mM NaH_2_PO_4_, 10 mM Tris–HCl, 8 M urea, pH 8.0) and stored at −70 °C. The cells were melted and sonicated 10 times for 10 s (200–300 V). The soluble protein fraction was obtained by centrifuging at 10 000 × *g* for 30 min at +4 °C.

Recombinant protein containing a His-tag at the amino terminus was purified on a Ni-NTA-agarose (Qiagen, Venlo, Netherlands) according to the manufacturer’s instructions. Briefly, the agarose was stirred with the soluble fraction of the *E. coli* supernatant for 2 h. The matrix was washed in the buffer (100 mM NaH_2_PO_4_, 10 mM Tris–HCl, 8 M urea, pH 6.3). Proteins were first eluted in the buffers containing 100 mM NaH_2_PO_4_, 10 mM Tris–HCl, 8 M urea, pH 5.9 and pH 4.5, respectively and continued in the buffer containing 100 mM NaH_2_PO_4_, 10 mM Tris–HCl, 8 M urea, 250 mM imidazole, pH 3.5.

Eluted proteins were concentrated with a Centricon Centrifugal Filter device (Millipore, Billerica, MA, USA) and separated with preparative SDS-PAGE. The gel was stained by 0.25 mM KCl [27] and TSC-22 was cut from the gel and eluted in a 3 ml buffer containing 20 mM Tris–HCl pH 8.0, 0.01 % SDS, 1 mM CaCl_2_ at +37 °C overnight, after which the buffer was changed into phosphate-buffered saline (PBS) by PD-10 column (GE Healthcare, Little Chalfont, United Kingdom). The antibody against TSC-22 was produced in rabbits by Davids Biotechnology (Regensburg, Germany). TSC-22 protein was injected three different times for immunization, and rabbit serum antibody was purified by precipitation and affinity purification. The specificity of the TSC-22 antibody was tested with the pure TSC-22 protein used as an antigen.

### Animals

Newborn, 2- to 4-day-old Sprague–Dawley rats of both sexes and male 2- to 3-month-old SD rats weighing from 250 to 300 g as well as 12-to-20-month-old spontaneously hypertensive rats (SHR) of the Okamoto-Aoki strain and age-matched Wistar-Kyoto (WKY) rats from the colony of the Centre of Experimental Animals at the University of Oulu, Finland, were used. The SHR strain was originally obtained from Møllegaards Avslaboratorium, Skensved, Denmark. All rats were kept in plastic cages with free access to tap water and regular rat chow in a room with a controlled 40 % humidity and a temperature of 22 °C. A 12 h light and 12 h dark environmental light cycle was maintained. All experimental protocols were approved by the Animal Use and Care Committee of the University of Oulu and the Provincial Government of Western Finland Department of Social Affairs and Health. The study conforms to the Guide for the Care and Use of Laboratory Animals published by the US National Institutes of Health.

### Experimental design in conscious normotensive and hypertensive rats

The Sprague Dawley rats were anesthetized with 0.26 mg/kg fentanyl citrate, 8.25 mg/kg fluanisone and 4.1 mg/kg midazolam i.p., and instrumented for vehicle and drug infusions as previously described [25]. The experiments were started in conscious animals by measuring mean arterial pressure (MAP) and heart rate for 30 min before baseline hemodynamic measurements were made. Then arginine vasopressin (AVP) (Peninsula Laboratories Europe) at a dose of 0.05 mg/kg per minute or vehicle (0.9 % NaCl) was infused i.v. at 37.5 ml/min for 30 min and 4 h.

Angiotensin II (Sigma Chemicals) (33 μg/kg/h) or vehicle (0.9 % NaCl) was administered through subcutaneously implanted osmotic minipumps (Alzet, Durect Corporation, Cupertino, CA, USA) in conscious rats for 6, 12 and 72 h, and 2 weeks as described previously [25]. In a separate series of experiments, vehicle (0.9 % NaCl), Ang II (33 μg/kg/h), losartan (400 μg/kg/h) [25] (a generous gift from Merck), or Ang II in combination with losartan was administered for 6 h.

In addition, osmotic minipumps (Alzet) releasing metoprolol (Sigma-Aldrich, St. Louis, MO, USA) (1.5 mg/kg/h) or losartan (400 μg/kg/h) were placed subcutaneously in WKY and SHRs, as described above. At the end of experiments, the rats were decapitated, the thoracic cavity was opened, and the heart was removed, left ventricular samples were blotted dry, weighed, frozen in liquid nitrogen and stored at −70 °C for further analysis.

### Experimental myocardial infarction

Myocardial infarction was produced by ligation of the left anterior descending coronary artery (LAD) [28]. The male Sprague–Dawley rats were anesthetized, a left thoracotomy and pericardial incision was made, and the LAD was ligated as previously described [29], producing left ventricular dilation, thinning of the anterior wall and hypertrophy of the posterior wall [29]. In a separate series of experiments, osmotic minipumps (Alzet) releasing metoprolol (Sigma-Aldrich) 1.5 mg/kg/h or losartan (400 μg/kg/h) were placed subcutaneously. The sham operated rats underwent the same surgical procedure without osmotic minipumps or ligation of LAD.

### Recombinant adenovirus vectors and adenoviral gene transfer *in vivo*

The recombinant adenoviruses containing the coding regions of the constitutively active MKK3b (RAdMKK3bE) and wild-type (WT) p38α (RAdp38α) genes driven by the cytomegalovirus immediate early promoter were generated as described [30]. Recombinant replication-deficient adenovirus RAd*lacZ*, which contains the *Escherichia coli* β-galactosidase (*LacZ*) gene, was used as a control virus. The recombinant adenovirus AxCAmTSC-22 (deposited by Kazunari Yokoyama) and AxCALacZ (deposited by Izumu Saito) were provided by the DNA Bank, RIKEN BioResource Center (Tsukuba, Japan) with the support of National Bio-Resources Project of Ministry of Education, Culture, Sports, Science and Technology Japan (MEXT). The recombinant adenovirus harbouring mouse TSC-22 (TGF beta 1-inducible gene) cDNA AxCAmTSC-22 (hereinafter referred to as TSC-22) and AxCALacZ (hereinafter referred to as LacZ) expressing beta-galactosidase, used as a control virus, were under the control of a CAG promoter [31].

Adenovirus-mediated gene transfer into the left ventricle was performed as a local intramyocardial injection, as previously described [32]. Male Sprague–Dawley rats weighing 250–300 g were anesthetized with medetomidine hydrochloride (Domitor 250 μg/kg, i.p.) and ketamine hydrochloride (Ketamine 50 mg/kg, i.p.). A left thoracotomy and pericardial incision was made. Recombinant TSC-22 adenovirus with a dose of 7 × 10^9^ pfu in a 100-μL volume was injected using a Hamilton precision syringe directly into the anterior wall of the left ventricle; the heart was then repositioned in the chest and the incision was closed. After the operation, the anaesthesia was partially antagonized with atipamezole hydrochloride (Antisedan 1.5 mg/kg, i.p.) and the rats were given buprenorphine hydrochloride (Temgesic, 0.05-0.2 mg/kg s.c.) for analgesia.

### Echocardiography

Transthoracic echocardiography was performed using a commercially available Acuson Ultrasound System (SequoiaTM 512) and a 15-MHz linear transducer (15 L8) (Acuson, Mountain View, CA, USA) as previously described [29, 33]. Echocardiographic measurements were performed by a skilled sonographer blinded to the treatments. After echocardiography, the animals were sacrificed, their hearts were weighed and the tissue samples were immersed in liquid nitrogen and stored at −70 °C for later analysis.

### Cell culture

Cell cultures of cardiac ventricular cells were isolated from 2-to-4 day old Sprague–Dawley rats as described [34]. The animals were killed by cervical dislocation and the ventricles were excised and cut into small pieces, and thereafter incubated 1–2 h at +37 °C in a solution containing 100 mM NaCl, 10 mM KCl, 1.2 mM KH_2_PO_4_, 4.0 mM MgSO_4_, 50 mM taurine, 20 mM glucose, 10 mM Hepes, 2 mg/ml collagenase type 2 (Worthington, Lakewood, NJ, USA), 2 mg/ml pancreatin (Sigma-Aldrich, St Louis, MO, USA) and 1 % penicillin–streptomycin (PS). The detached cells were collected after incubation by centrifuging at 900 rpm for 5 min. The bottom layer of the pellet containing undamaged isolated cardiomyocytes was plated on 35 mm dishes at a density of 1.4 × 10^5^/cm^2^. The cells were cultured overnight in Dulbecco’s modified Eagle’s medium nutrient mixture F-12 ham with L-glutamine (DMEM/F12) containing 10 % foetal bovine serum (FBS) and 1 % PS. Thereafter the cells were cultured in complete serum free medium (CSFM) containing DMEM/F12, 2.5 mg/ml bovine serum albumin, 1 μM insulin, 5.64 μg/ml transferrin, 32 nM selenium, 2.8 mM Na-pyruvate, 0.1 nM T_3_ and 1 % PS. The cells were infected with adenoviruses 24 h after the cells were plated at the virus concentration of 2, 4 or 8 MOI (multiplicity of infection). Fresh CSFM was changed 24 h after the infection and cells were cultured in all 72 h before being washed twice with ice-cold PBS and stored at −70 °C. Endothelin-1 (ET-1) treatment was performed as described before [35, 36].

### Isolation and analysis of RNA

Total RNA from tissue samples was isolated by the guanidine thiocyanate-CsCl method and from cultured cardiomyocytes with TRIzol Reagent according to the manufacturer’s protocol (Invitrogen, CA, USA) by using the Phase Lock Gel system (Eppendorf AG, Hamburg, Germany) as previously described [37]. For Northern Blot analyses, 20 μg samples of RNA from LV tissue were separated on agarose–formaldehyde gel electrophoresis and transferred to nylon membranes (Osmonics Inc.). PCR-amplified Northern probes corresponding following genes: rat TSC-22 and 18S were random primer-labelled with [α^32^P]dCTP, and the membranes were hybridized and washed as described previously [25]. The hybridization signals were normalized to 18S RNA quantified from the same samples.

Rat TSC-22, Atrial natriuretic peptide (ANP), B-type natriuretic peptide (BNP), interleukin-6 (IL-6), connective tissue growth factor (CTGF), plasminogen activator inhibitor-1 (PAI-1), fibronectin-1 (Fn-1), collagen type Iα1 (Col1a1) and type IIIα1 (col3a1), Bcl-*2*-associated X protein (Bax2), B-cell lymphoma 2A (Bcl2A) and 18S mRNA levels were measured by real time quantitative PCR using TaqMan chemistry on an ABI 7300 Sequence Detection System (Applied Biosystems, Foster City, CA, USA) as described previously [37]. The sequences of the forward and reverse primers and fluorogenic probes for RNA are given in Table 1. The results were normalized to 18S RNA quantified from the same samples.

**Table 1:** Sequences of primers and fluorogenic probes used in real time quantitative RT-qPCR analysis (sequences 5′ to 3′)

Gene	Forward Primer	Reverse Primer	Fluorogenic Probe
TSC-22	GCAGGAGAACAATC	TGCAGCTGGG	CCAGTCCGGAG
	TGTTGAAGAC	CCTGAAAC	CAGCTCGCC
ANP	GAAAAGCAAACTG	CCTACCCCCG	TCGCTGGCCCT
	AGGGCTCTG	AAGCAGCT	CGGAGCCT
BNP	TGGGCAGAAGATAG	ACAACCTCAG	CGGCGCAGTCA
	ACCGGA	CCCGTCACAG	GTCGCTTGG
IL-6	ATATGTTCTCAGGG	TGCATCATCGC	CAGAATTGCCATTGCA
	AGATCTTGGAA	TGTTCATACAA	CAACTCTTTTCTCA
CTGF	CGCCAACCGCAAGA	CACGGACCCA	CACTGCCAAAGA
	TTG	CCGAAGAC	TGGTGCACCCTG
PAI-1	GCTGACCACAGCAG	GTGCCCCTCTCA	CCCGGCAGCAGAT
	GGAAA	CTGATATTGAA	CCAAGATGCTAT
col1a1	CCCCTTGGTCTTGG	GCACGGAAAC	CTTTGCTTCCCAGATGT
	AGGAA	TCCAGCTGAT	CCTATGGCTATGATG
Fn-1	GCGAGGCAGGATC	CCAATCTTGTA	ACCATTGCAAAT
	AGCTG	GGACTGACCCC	CGCTGCCATGAA
Bax2	TCCCCCCGAGAGG	CCCAGTTGAAG	CCGGGTGGCAGCT
	TCTTCT	TTGCCATCA	GACATGTTTG
Bcl2A	GGAACTATATGG	AGGCTGAGCA	CGACCTCTGTTTG
	CCCCAGCAT	GGGTCTTCAG	ATTTCTCCTGGCTGTC
col3a1	AGCTGGCCTTCCTC	GCTGTTTTTGCAG	TTCCAGCCGG
	AGACTTC	TGGTATGTAATG	GCCTCCCAG
18S	TGGTTGCAAAGCT	AGTCAAATTA	CCTGGTGGTGC
	GAAACTTAAAG	AGCCGCAGGC	CCTTCCGTCA

*TSC-22* transforming growth factor beta-stimulated clone 22; *ANP* atrial natriuretic peptide; *BNP* B-type natriuretic peptide; *IL-6* interleukin-6; *CTGF* connective tissue growth factor; *PAI-1* plasminogen activator inhibitor-1; *col1a1* collagen type Iα1; *Fn-1* fibronectin-1; *Bax2* Bcl-*2*-associated X protein; *Bcl2A* B-cell lymphoma 2A; *col3a1* collagen type IIIα1

### Protein extraction

The tissue was broken in liquid nitrogen, and homogenized in a lysis buffer consisting of 20 mmol/L Tris (pH 7.5), 10 mmol/L NaCl, 0.1 mmol/L EDTA, 0.1 mmol/L EGTA, 1 mmol/L β-glycerophosphate, 1 mmol/L Na_3_VO_4_, 2 mmol/L benzamidine, 1 mmol/L PMSF, 50 mmol/L NaF, 1 mmol/L DTT and 10 μg/mL of each leupeptin, pepstatin and aprotinin as previously described [32, 37]. The tissue homogenates were centrifuged at 2000 rpm in +4 °C for 1 min. To separate the total protein fraction, 5× NEB was added to the tissue homogenate, following by centrifugation at 12,500 rpm for 20 min. The supernatant was stored in −70 °C until assayed.

To extract total protein from cultured cardiomyocytes, the cells were scraped in 100–150 μl of 1× NEB buffer containing protease-inhibitor cocktail (1:100 volume), phosphatase-inhibitor cocktail (1:100 volume) and 1 mM dithiothreitol (DTT) (1:1000 volume). Cells were vortexed for 20 s and centrifuged 12,500 rpm for 20 min at +4 °C. The supernatant was collected as the total protein fragment. The entire procedure was carried out at +4 °C. The supernatant was stored in −70 °C until assayed. Protein concentrations were determined by colorimetric assay (Bio-Rad Laboratories, Hercules, CA, USA).

### Western blotting

Western blots were performed as previously described [35]. Briefly, 15–60 μg of protein was loaded onto a SDS-PAGE gel and transferred to nitrocellulose filters. The membranes were blocked in 5 % non-fat milk and incubated with anti-TSC-22 polyclonal antibody (dilution 1:500 in 0.5 % milk-TBS-Tween) overnight. After incubation with secondary anti-rabbit antibody (Cell Signaling Technology, Danvers, MA, USA) at the dilution of 1:2000, the protein amount was detected by ECL Plus (GE Healthcare Life Sciences). For Odyssey detection, the membranes were blocked in Odyssey Blocking Buffer – TBS (1:1) and incubated with anti-TSC-22 polyclonal antibody or anti-GAPDH (glyceraldehyde-3-phosphate dehydrogenase) (Millipore, MA, USA) overnight. After incubation with secondary Alexa Fluor anti-rabbit or anti-mouse antibody (Invitrogen), the protein levels were detected using Odyssey Infrared Detection.

### Histological analysis

For immunohistochemical analysis, the hearts were fixed in 10 % buffered formalin solution. Transverse sections of the left ventricle were embedded in paraffin, and 5-μm-thick sections were cut from the midsection of the heart, at the level of the papillary muscles. Sections were deparaffinised in xylene and dehydrated in graded ethanol, and to identify cellular localization of TSC-22, incubated with specific anti-TSC-22 polyclonal antibody at a dilution of 1:500. The primary antibody was detected by a peroxidase conjugated Dako REAL™ EnVision™ Detection System, Peroxidase/DAB kit (Dako, Glostrup, Denmark) according to the manufacturer’s instructions, and the samples were counterstained with hematoxylin and eosin. All measurements were performed by persons blinded to the treatments.

### Human LV tissue samples

LV samples were obtained from explanted human hearts from 4 patients with cardiomyopathy (2 dilated, 1 idiopathic, 1 hypertrophic) undergoing heart transplantation. Non-diseased donor hearts unsuitable for transplant were used to obtain control myocardial tissue (*n* = 2). All tissues were obtained with signed informed consent of the subjects. The project was approved by the local Ethics Committee (Ethical permission number: 5012-0/2011-EKU142/PI/11–Semmelweis University, Hungary) and conducted in accordance with the guidelines of the Declaration of Helsinki.

### Statistics

Statistical analysis was performed by unpaired Student’s *t*-test or One-way Analysis of variance (ANOVA) followed by a least significant difference (LSD) post hoc test for multiple comparisons. A value of *P* <0.05 was considered to be statistically significant. The results are expressed as mean ± standard error of the mean (SEM).

## Results

### Cardiac remodelling in chronic hypertension and after experimental myocardial infarction modulates TSC-22 gene expression

**Fig. 1:**
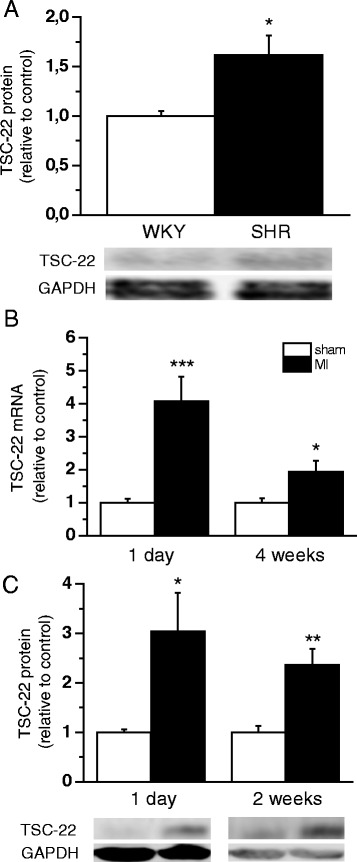
Effects of cardiac remodelling on chronic hypertension and, after experimental myocardial infarction (MI), on transforming growth factor beta-stimulated clone 22 (TSC-22) expression. **a** Left ventricular (LV) tissue TSC-22 total protein levels were analysed by Western blot from 12 and 20 months old Wistar Kyoto (WKY) and spontaneously hypertensive rat (SHR) hearts (*n* = 5). Open bars represent WKY and solid bars SHR *P<0.05 versus WKY (Student’s *t*-test). The effect of MI on LV tissue TSC-22 mRNA levels (**b**) measured by Northern blot or RT-PCR and TSC-22 protein levels (**c**) analysed by Western blot (*n* = 6−8). Open bars represent sham and solid bars MI *P<0.05, **P <0.01, ***P <0.001 versus sham (Student’s *t*-test). The results are expressed as mean ± SEM. Representative Western blots are shown

DNA-microarray studies have previously shown that TSC-22 mRNA levels are elevated both in old SHRs [33] and after experimental MI in rats [26]. First, we examined TSC-22 protein levels, which were higher in 12-to-20-month-old SHR (1.6-fold, *P* <0.05) compared with age-matched normotensive WKY rats (Fig. 1a). Then we investigated the induction of TSC-22 in the heart more carefully after MI. Similarly to gene expression profiles of Stanton et al. [26], LV TSC-22 mRNA levels were up-regulated 4 weeks after MI (Fig. 1b). In addition, a significant increase in mRNA levels was seen already on day 1 following MI when compared to sham-operated animals (4.1-fold, *P* <0.001), indicating that TSC-22 induction in the post-infarction cardiac remodelling process is rapid and persistent. Similar profiles to mRNA, in the degree and the time course of the induction, were seen in the protein up-regulation, as assessed by Western blot analysis (Fig. 1c). TSC-22 mRNA and proteins were also found to be expressed in the left ventricle of control human hearts and in patients with cardiomyopathy, but there were no changes in the expression levels between failing and control hearts (data not shown).

### TSC-22 gene expression is induced in the adult heart with acute pressure overload

**Fig. 2:**
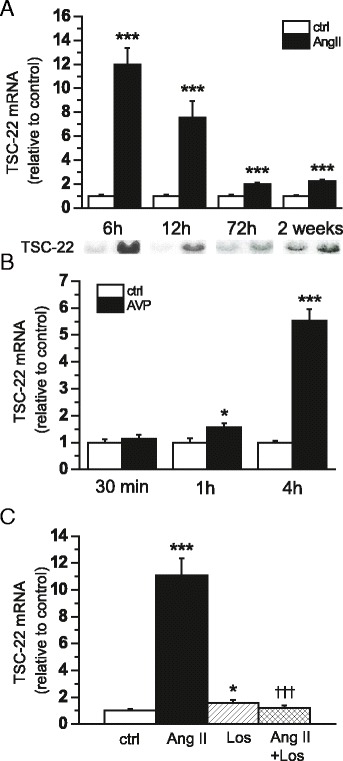
The effect of acute and chronic pressure overload on transforming growth factor beta-stimulated clone 22 (TSC-22) gene expression. **a** The effect of angiotensin II (Ang II) administration (33 μg/kg/h) on left ventricular (LV) TSC-22 mRNA levels assessed by Northern blot analysis in rats (*n* = 6−8). Open bars represent control and black bars Ang II ***P <0.001 versus control (Student’s *t*-test). **b** TSC-22 mRNA levels after arginine vasopressin (AVP) infusion (*n* = 9). Open bars represent control and solid bars AVP *P <0.05, ***P <0.001 versus control (Student’s *t*-test). **c** The effect of Ang II and AT_1_-receptor antagonist losartan (Los) on LV TSC-22 mRNA levels at 6 h (*n* = 5−8). *P <0.05, ***P <0.001 versus control. ^ƗƗƗ^ *P* <0.001 versus Ang II (one-way ANOVA followed by least significance difference post hoc test). The results are expressed as mean ± SEM. Representative Northern blots are shown

Next, we studied the effect of acute and chronic pressure overload on LV TSC-22 expression. Ang II increased TSC-22 mRNA levels from 6 h to 2 weeks, the greatest increase (12.0-fold, *P* <0.001) being observed after 6 h of Ang II infusion (Fig. 2a). Accordingly, *in vivo* administration of AVP induced a small, but significant increase in TSC-22 already after 1 h, and at 4 h, TSC-22 mRNA levels were markedly elevated (5.5-fold, *P* <0.001) (Fig. 2b). Moreover, the Ang II type 1-receptor antagonist losartan completely abolished the changes in TSC-22 gene expression induced by Ang II in the left ventricle (Fig. 2c).

### The effect of long-term treatment of heart failure with metoprolol and hypertension with metoprolol or losartan on TSC-22 expression

**Fig. 3:**
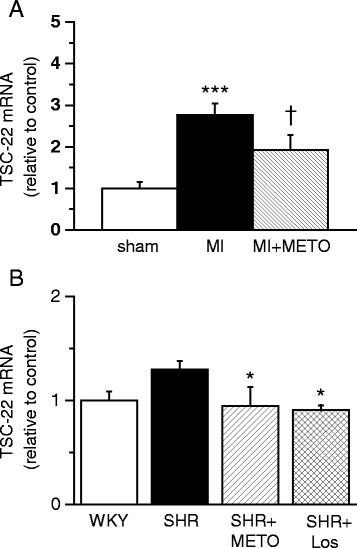
The effect of long-term treatment of heart failure and hypertension with metoprolol on transforming growth factor beta-stimulated clone 22 (TSC-22) expression. **a** Left ventricular (LV) TSC-22 mRNA levels measured by RT-PCR from rats 2 weeks after myocardial infarction (MI) and after metoprolol (METO) treatment post-MI (*n* = 7-8). ***P <0.001 versus sham. ^Ɨ^ *P* <0.001 versus MI (one-way ANOVA followed by least significance difference post hoc test). **b** LV TSC-22 mRNA levels from 12-month-old Wistar Kyoto (WKY) rats, untreated spontaneously hypertensive rats (SHR) and SHRs treated with metoprolol (METO) or losartan (Los) for 14 days (*n* = 5-6). *P <0.05 versus untreated SHR (one-way ANOVA followed by least significance difference post hoc test). The results are expressed as mean ± SEM

Next, we aimed to characterize whether TSC-22 expression changes in response to a treatment with a beta-blocker metoprolol or losartan in left ventricular hypertrophy or heart failure. First, we characterized the LV TSC-22 gene expression in rats treated with metoprolol during the remodelling in post-MI [38]. Post-infarction treatment with metoprolol improved LV systolic and diastolic function and attenuated LV remodelling as previously described [38]. After MI, LV TSC-22 mRNA levels were increased 2.8-fold (*P* < 0.001) (Fig. 3a). Metoprolol administration for 2 weeks via osmotic minipumps reduced LV TSC-22 mRNA levels by 32 % (*P* <0.05). Next, we infused metoprolol for two weeks in 12-month-old SHRs. Metoprolol treatment lowered the heart beat and reduced enlargement of LV end-diastolic dimension (7.7 ± 0.1 in SHR vs. 8.5 ± 0.1 in SHR treated with metoprolol; *P* <0.001). Again, metoprolol reduced significantly the TSC-22 mRNA levels in metoprolol-treated SHRs when compared with untreated SHRs (Fig. 3b). A similar decrease in TSC-22 gene expression was also seen after 2 weeks of losartan infusion in SHRs (Fig. 3b), when reduced thickness of the LV wall was seen in losartan-treated SHRs compared to untreated SHRs (interventricular septal thickness −15.4 %, *P*<0.001 and left ventricular posterior wall thickness in diastole −18.2 %, *P* <0.05).

### Signalling pathways regulating TSC-22 gene expression

**Fig. 4:**
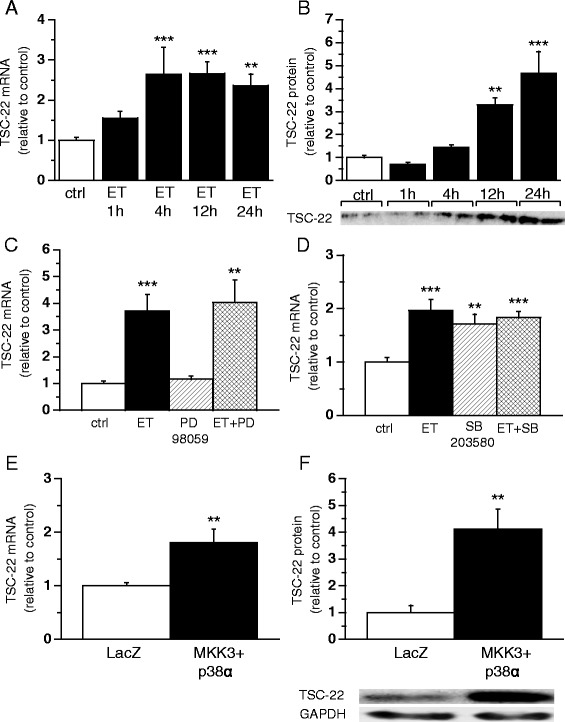
Signalling pathways regulating transforming growth factor beta-stimulated clone 22 (TSC-22) gene expression. Cultured neonatal rat ventricular myocytes (NRVMs) were treated with endothelin-1 (ET, 100 nM) for 1, 4, 12 and 24 h. **a** TSC-22 mRNA levels were measured by RT-PCR (*n* = 9−12 from three independent experiments) **b**) and TSC-22 total protein levels were determined by Western blot (*n* = 4). Open bars represent control and solid bars ET **P <0.01, ***P <0.001 versus control (one-way ANOVA followed by least significance difference post hoc test). TSC-22 mRNA levels in NRVMs exposed to kinase inhibitor (**c**) PD98059 (10 μM) or **d**) SB203580 (10 μM), and treated with 100 nM ET for 1 h (*n* = 8−9, from three independent experiments). **P <0.01, ***P <0.001 versus control (Student’s *t*-test). TSC-22 **e**) mRNA levels measured by Northern blot and **f**) total protein levels analysed by Western blot in left ventricle (LV) of Sprague–Dawley (SD)-rats after adenovirus induced MKK3bE and WT p38alpha overexpression at day 3 (*n* = 7–10). Open bars represent LacZ and solid bars MKK3bE and WT p38alpha **P <0.01 versus LacZ (Student’s *t*-test). The results are expressed as mean ± SEM. Representative Western blots are shown

We determined the cellular mechanisms involved in the regulation of TSC-22 gene expression by testing the effect of hypertrophic agonist ET-1 on TSC-22 mRNA levels in neonatal rat ventricular myocytes (NRVMs). ET-1 (100 nM) significantly increased TSC-22 gene expression with maximal mRNA levels at 12 h (2.7–fold, *P* <0.001) (Fig. 4a) and protein levels at 24 h (4.7-fold, *P*<0.001) (Fig. 4b). Next, we investigated intracellular signalling pathways mediating ET-1 induced TSC-22 gene expression by focusing on MAPKs, since all MAPK pathways are key signalling routes known to be activated in the heart in response to hypertrophic stimuli including ET-1 [4]. The neonatal rat ventricular myocytes were exposed to kinase inhibitors SB203580 (10 μM) or PD98059 (10 μM), and the cells were treated with 100 nM ET-1 for 1 h. Neither PD98059 (Fig. 4c) nor SB203580 (Fig. 4d) had significant effect on ET-1 –induced increase in TSC-22 mRNA levels in NRVMs. However, the p38α and p38β MAPK inhibitor SB203580 significantly increased TSC-22 mRNA levels in NRVMs (Fig. 4d).

Finally, in order to determine whether p38 MAPKs regulate cardiac TSC-22 gene expression *in vivo*, adenoviruses expressing constitutively active MKK3bE and WT p38alpha to cause p38 MAPK-overexpression were injected into the LV free wall of adult Sprague–Dawley rats. Adenoviral p38 MAPK overexpression significantly increased both TSC-22 mRNA (1.8-fold, *P* <0.01) and protein levels (4.1-fold, *P* <0.01) at day 3 following gene transfer (Fig. 4e-f).

### The role of TSC-22 in regulating cardiac gene expression

**Fig. 5:**
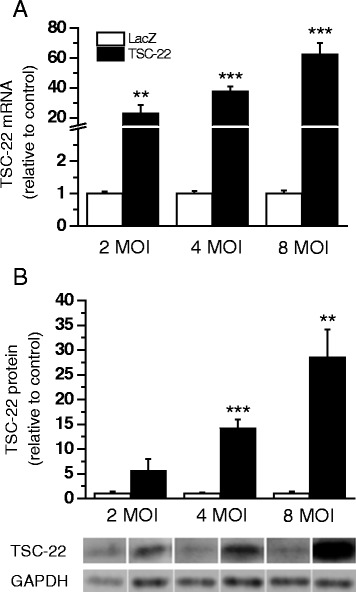
Effects of transforming growth factor beta-stimulated clone 22 (TSC-22) overexpression in cultured neonatal rat ventricular myocytes (NRVMs). TSC-22 **a**) mRNA levels measured by RT-PCR (*n* = 8–12) or **b**) total protein levels determined by Western blot analysis (*n* = 4) from cultured NRVMs transduced with TSC-22 or LacZ adenovirus for 48 h at the virus amount of 2 MOI, 4 MOI and 8 MOI. **P <0.01, ***P <0.001 versus LacZ (Student’s *t*-test). The results are expressed as mean ± SEM. Representative Western blots are shown

In order to study the direct myocardial effects of TSC-22 in regulating cardiac gene expression, we established an *in vivo* gene transfer protocol to locally increase TSC-22 levels in the adult rat heart. First, the efficiency and functionality of the adenovirus gene transfer was studied by transducing the cardiomyocyte cell cultures with TSC-22 adenovirus. Adenovirus transduction markedly increased both TSC-22 mRNA and protein levels at the virus amounts of 2, 4 and 8 MOI in a dose-dependent manner (Fig. 5a, b).

**Fig. 6:**
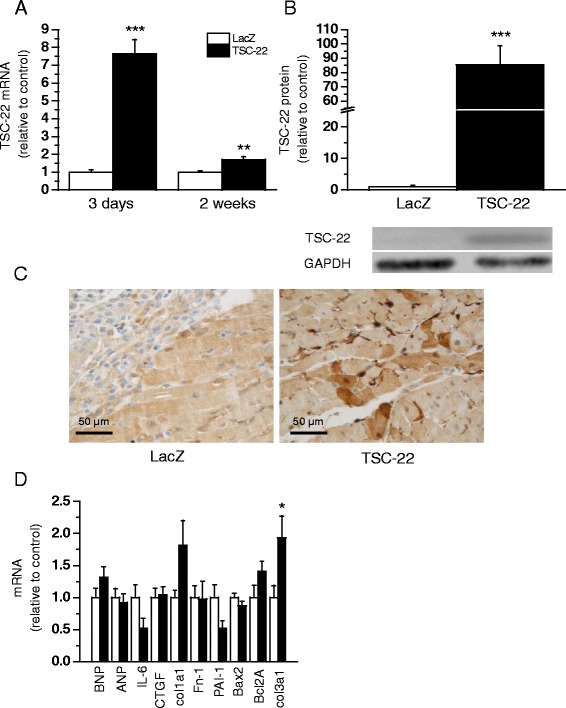
Effects of adenovirus-mediated transforming growth factor beta-stimulated clone 22 (TSC-22) gene delivery in normal rat hearts. TSC-22 **a** mRNA levels measured by RT-PCR 3 days or 2 weeks (*n* = 5–8) and **b** total protein levels analysed by Western blot (*n* = 8) 3 days after gene delivery in left ventricular (LV) tissue samples of Sprague–Dawley (SD)-rat hearts. **c** Localization of TSC-22 at cellular level was studied by immunohistochemical staining against TSC-22 at day 3 after gene transfer. **d** mRNA levels of B-type natriuretic peptide (BNP), atrial natriuretic peptide (ANP), interleukin-6 (IL-6), connective tissue growth factor (CTGF), collagen type Iα1 (col1a1), fibronectin-1 (Fn-1), plasminogen activator inhibitor-1 (PAI-1), Bcl-*2*-associated X protein (Bax2), B-cell lymphoma 2A (Bcl2A) and collagen type IIIα1(col3a1) measured by RT-PCR from LV of SD-rat hearts 2 weeks after gene delivery (*n* = 5). *P <0.05, **P <0.01, ***P <0.001 versus LacZ (Student’s *t*-test). The results are expressed as mean ± SEM. Representative Western blots are shown

Next, the effect of TSC-22 gene delivery on cardiac function was studied *in vivo* [32]. TSC-22 mRNA levels were increased in response to adenoviral TSC-22 overexpression at three days up to 2 weeks (7.7-fold, *P* <0.001 and 1.7-fold, *P*<0.01, respectively) following injections (Fig. 6a). Similarly, the induction in TSC-22 protein levels was seen 3 days after TSC-22 gene transfer (Fig. 6b). TSC-22 gene transfer had no significant effect on LV dimensions or cardiac function as assessed by echocardiography (Table 2).

**Table 2:** Effect of intramyocardial TSC-22 gene delivery on cardiac function in the normal adult rat heart

Variable	Group	TSC-22	TSC-22
		3 days	2 weeks
Interventricular septum diastole (mm)	LacZ	1.9 ± 0.1	2.0 ± 0.1
	TSC-22	2.1 ± 0.1	2.0 ± 0.1
Interventricular septum systole (mm)	LacZ	2.6 ± 0.1	2.5 ± 0.2
	TSC-22	2.7 ± 0.2	2.8 ± 0.2
Left ventricle diastole (mm)	LacZ	7.6 ± 0.3	8.6 ± 0.1
	TSC-22	7.2 ± 0.3	8.7 ± 0.2
Left ventricle systole (mm)	LacZ	5.1 ± 0.3	6.1 ± 0.3
	TSC-22	4.7 ± 0.3	6.1 ± 0.3
Posterior wall distole (mm)	LacZ	2.3 ± 0.2	1.6 ± 0.1
	TSC-22	2.0 ± 0.3	1.5 ± 0.1
Posterior wall systole (mm)	LacZ	2.1 ± 0.2	2.3 ± 0.2
	TSC-22	2.1 ± 0.1	2.3 ± 0.1
Ejection fraction (%)	LacZ	66.7 ± 2.3	58.2 ± 4.5
	TSC-22	69.1 ± 3.0	62.8 ± 3.0
Fractional shortening (%)	LacZ	32.8 ± 1.7	27.4 ± 2.7
	TSC-22	34.7 ± 2.5	30.3 ± 2.0
IVRT	LacZ	no data	24.8 ± 2.2
	TSC-22	no data	25.0 ± 1.6
E/A-ratio	LacZ	no data	5.1 ± 0.8
	TSC-22	no data	2.7 ± 0.6*
		*n* = 7–8	*n* = 5

Adenoviral gene construct expressing TSC-22 or LacZ were injected into LV free wall and echocardiographic measurements were performed at 3 days and 2 weeks after gene transfer. The results are expressed as mean ± SEM. *P < 0.05 versus LacZ (Student’s *t*-test). IVRT, isovolumic relaxation time

To evaluate the localization of TSC-22, we performed immunohistochemical studies of TSC-22 overexpressing hearts and LacZ injected control hearts. At 3 days, TSC-22 immunohistochemistry showed a very strong cytoplasmic staining that localized to the endothelium of myocardial capillaries at the injection site (Fig. 6c). In addition, some myocytes in the inflammation area of TSC-22 overexpressing hearts displayed cytoplasmic and nuclear positivity in TSC-22 immunostaining. In control hearts, there was also some positivity in the myocytes of inflammation areas, but this was weaker than in TSC-22 overexpressing hearts, and no remarkable endothelial positivity could be seen.

Next, we determined the influence of TSC-22 gene transfer on expression of several well-established markers of cardiac hypertrophy, fibrosis and apoptosis. TSC-22 gene delivery did not have an influence on expression levels of natriuretic peptides (ANP and BNP), inflammatory and ECM modulatory factors (IL-6, CTGF, PAI-1, Fn-1, col1a1) or apoptosis (Bax2, Bcl2A). The only statistically significant difference between the TSC-22 and LacZ-treated groups was in increased col3a1 gene expression levels 2 weeks after TSC-22 gene delivery (Fig. 6d).

## Discussion

TGF-β is one of the key mediators both in the embryonic development of the heart and a number of cardiac diseases such as post-MI remodelling and pressure overload hypertrophy [7, 8]. TGF-β activates transcription factors such as SMAD proteins, and TSC-22. TSC-22 is suggested to have functions in controlling tumorigenesis, apoptosis, cholesterol homeostasis, cardiac fibrosis as well as development and differentiation processes [14–23]. Overall in this study, we extended knowledge of the role of TSC-22 in the heart. We found that TSC-22 gene expression is rapidly up-regulated by multiple hypertrophic stimuli and post-MI remodelling in the heart. We showed that both losartan and metoprolol treatments reduced left ventricular TSC-22 gene expression levels. We also observed that growth-promoting factor ET-1, activated TSC-22 mRNA and protein expression in cultured neonatal ventricular myocytes, and that p38MAPK signalling regulated cardiac TSC-22 expression *in vivo*. By using local intramyocardial adenovirus-mediated gene delivery, we evaluated the effect of TSC-22 overexpression on cardiac gene expression, showing that TSC-22 appears to have a role in regulating *col3a1* gene expression in the heart.

In the present study, we showed that TSC-22 gene expression was rapidly up-regulated in response to AVP- and Ang II-infused pressure overload, mimicking the expression patterns of immediate-early genes such as *Egr-1, c-jun* and *c-fos* in response to hemodynamic stress [39]. Elevated mRNA levels have previously been described by chronic pressure overload in the left ventricle of 20-month-old SHRs and at 2 to 16 weeks after experimental myocardial infarction in the rat [26, 33]. Altogether, the data implies that TSC-22 is up-regulated throughout the time course of cardiac remodelling suggesting it has a constitutive role in activating cardiac remodelling-related gene expression. However, although TSC-22 was expressed in the human heart both at mRNA and protein level, it was not up-regulated in patients with end-stage heart failure. Even though we cannot rule out the species difference (human vs. rodent), one possibility is that elevated amounts of TSC-22 are needed for controlling the transcriptional response of the active remodelling process and in the functional deterioration but no longer at the failing stage.

Cardiac remodelling after myocardial infarction and in hypertensive hypertrophy is an important target for drug treatment. In our study, both beta-blocker and ATR-antagonist treatment reduced left ventricular TSC-22 gene expression levels. Several studies suggest that there is an association between TGF-β1 and both the renin-angiotensin-aldosterone and the beta-adrenergic systems [7, 8, 40]. Ang II induces TGF-β1 expression in cardiac myocytes and fibroblasts *in vitro* [7, 8, 40]. TGF-β1 is implicated in AngII-induced cardiac hypertrophy and beta-adrenergic signalling in the heart *in vivo* [41–43]. Furthermore, TGF-β1 has been shown to modulate beta-adrenergic receptors in various cell types [8, 44]. However, in TGF-β1 transgenic mice chronic β-adrenoceptor blockade was able to reverse the structural and functional changes of the heart, but treatment with the AT1-receptor antagonist losartan was not [45]. In our study, using the rat chronic pressure overload model, both betablocker metoprolol and AT1R antagonist losartan caused a reduction of TSC-22 gene expression, which could be due to the beneficial effects of these drugs on the development of cardiac fibrosis [46–48]. In SHR ventricular remodelling, cardiac hypertrophy is associated with an increase in fibrosis [49, 50], and it has been shown that endogenous AngII, mediated by AT1 receptors, plays a role in the formation of fibrosis e.g. by stimulating of TGF-β1-synthesis [7, 8, 40]. The effect of the AT1 receptor antagonist on TSC-22 gene expression was also observed in response to acute pressure overload, which can also be due to the inhibition of an Ang II-induced increase in blood pressure. So, it is likely that the effects of losartan on TSC-22 expression are explained by multiple mechanisms.

Almost nothing is known about the mechanisms and signalling pathways that regulate cardiac TSC-22 gene expression. We observed that ET-1 activated TSC-22 mRNA and protein expression in cultured neonatal ventricular myocytes. In our study, no ERK inhibitor PD98059 or p38 inhibitor SB203580 had an effect on ET-1-mediated activation of TSC-22 gene expression in cardiac myocytes. In fact, SB203580 alone was able to increase TSC-22 levels significantly. In addition, the role of p38 MAPK as a regulator of TSC-22 expression was studied in the adult heart *in vivo*. Since adenovirus-mediated overexpression of p38 MAPK *in vivo* increased TSC-22 gene expression, our results suggest that cardiac remodelling after MI and in hypertensive hypertrophy upregulates TSC-22 expression in the heart via the p38 MAPK pathway. However, the results are difficult to interpret because the p38 inhibitor SB203580 did not alter ET-1 induced TSC-22 up-regulation *in vitro*, which might be due to the fact that SB203580 by itself at the dose used in cell culture was able to increase TSC-22 gene expression. Although expression information alone is not sufficient to establish firm functional associations, the increased expression in the p38 MAPK overexpressed hearts exhibiting fibrosis and infiltration of inflammatory cells [32] supports previous findings that the TSC-22 may contribute to the development of cardiac fibrosis [13].

A key finding of our present study is that TSC-22 gene delivery in the adult rat heart regulates collagen synthesis. TSC-22 overexpression *in vivo* increased *col3a1* gene expression significantly, and there was a similar trend in col1a1 mRNA levels. In previous studies, lentivirus-mediated overexpression of TSC-22 enhanced the TGFβ -induced expression of a- collagen I in cultured rat fibroblasts [13] and in kidney cells, TSC-22 was shown to regulate col1a2 [51]. Collagens type I and III are major constituents of cardiac ECM [52], and in fibrosis e.g. the synthesis of collagen and degradation of collagen scaffold are altered. Our data together with previous findings [13, 51] suggest that TSC-22 has a role in regulating collagen synthesis during cardiac remodelling. Contrary to a previous study using neonatal rat cardiac fibroblasts [13], TSC-22 overexpression did not have any effect on PAI-1 or fibronectin, which may due to the cell type specific differences in the regulation of gene expression. Similarly to a previous study [53], TSC-22 was located in both nuclear and cytoplasmic regions, indicating that TSC-22 might have an effect on cardiac structure by regulating ECM-modulating genes at transcriptional level.

To summarize, we studied the TSC-22 expression during cardiac modelling in post-MI and hypertensive hypertrophy. We noticed that TSC-22 expression is rapidly increased in response to pressure overload and myocardial injury, and elevated expression levels are sustained until the failing phase of heart failure. Our analysis provides new insights into the transcriptional regulation of cardiac remodelling by showing that TSC-22 appears to have a role in regulating collagen 3a1 expression in the heart *in vivo*. In accordance with previous findings, our data suggests that TSC-22 could be a target for preventing the formation of cardiac fibrosis.

## Conclusions

In conclusion, TSC-22 expression in the rat heart is regulated by multiple hypertrophic stimuli and post-MI remodelling. Our data suggests that TSC-22 might regulate collagen synthesis in the heart *in vivo* and could be a potential target for fibrosis-preventing therapies.

## Abbreviations

AngII: Angiotensin II
col: Collagen
ET-1: Endothelin-1
LV: Left ventricle
MAPK: Mitogen activated kinase
MI: Myocardial infarction
NRVM: Neonatal rat ventricular myocytes
SHR: Spontaneously hypertensive rat
TGF-β: Transforming growth factor –β
TSC-22: TGF-β-stimulated clone 22
WKY: Wistar Kyoto

## References

[1] Mann DL, Barger PM, Burkhoff D (2012). Myocardial recovery and the failing heart: myth, magic, or molecular target?. J Am Coll Cardiol.

[2] Go AS, Mozaffarian D, Roger VL, Benjamin EJ, Berry JD, Blaha MJ (2014). Heart disease and stroke statistics--2014 update: a report from the American Heart Association. Circulation.

[3] Oka T, Xu J, Molkentin JD (2007). Re-employment of developmental transcription factors in adult heart disease. Semin Cell Dev Biol.

[4] Rose BA, Force T, Wang Y (2010). Mitogen-activated protein kinase signaling in the heart: angels versus demons in a heart-breaking tale. Physiol Rev.

[5] Akazawa H, Komuro I (2003). Roles of cardiac transcription factors in cardiac hypertrophy. Circ Res.

[6] Dobaczewski M, Chen W, Frangogiannis NG (2011). Transforming growth factor (TGF)-beta signaling in cardiac remodeling. J Mol Cell Cardiol.

[7] Bujak M, Frangogiannis NG (2007). The role of TGF-beta signaling in myocardial infarction and cardiac remodeling. Cardiovasc Res.

[8] Rosenkranz S (2004). TGF-beta1 and angiotensin networking in cardiac remodeling. Cardiovasc Res.

[9] Liu G, Ding W, Neiman J, Mulder KM (2006). Requirement of Smad3 and CREB-1 in mediating transforming growth factor-beta (TGF beta) induction of TGF beta 3 secretion. J Biol Chem.

[10] Shibanuma M, Kuroki T, Nose K (1992). Isolation of a gene encoding a putative leucine zipper structure that is induced by transforming growth factor beta 1 and other growth factors. J Biol Chem.

[11] Kester HA, Blanchetot C, Hertog J, Van der Saag PT, Van der Burg B (1999). Transforming growth factor- beta -stimulated clone-22 is a member of a family of leucine zipper proteins that can homo- and heterodimerize and has transcriptional repressor activity. J Biol Chem.

[12] Choi SJ, Moon JH, Ahn YW, Ahn JH, Kim DU, Han TH (2005). Tsc-22 enhances TGF-beta signaling by associating with Smad4 and induces erythroid cell differentiation. Mol Cell Biochem.

[13] Yan X (2011). TSC-22 promotes transforming growth factor beta-mediated cardiac myofibroblast differentiation by antagonizing Smad7 activity. Mol Cell Biol.

[14] Dobens LL, Hsu T, Twombly V, Gelbart WM, Raftery LA, Kafatos FC (1997). The Drosophila bunched gene is a homologue of the growth factor stimulated mammalian TSC-22 sequence and is required during oogenesis. Mech Dev.

[15] Kawamata H, Nakashiro K, Uchida D, Hino S, Omotehara F, Yoshida H (1998). Induction of TSC-22 by treatment with a new anti-cancer drug, vesnarinone, in a human salivary gland cancer cell. Br J Cancer.

[16] Kawamata H, Fujimori T, Imai Y (2004). TSC-22 (TGF-beta stimulated clone-22): a novel molecular target for differentiation-inducing therapy in salivary gland cancer. Curr Cancer Drug Targets.

[17] Ohta S, Yanagihara K, Nagata K (1997). Mechanism of apoptotic cell death of human gastric carcinoma cells mediated by transforming growth factor beta. Biochem J.

[18] Shostak KO, Dmitrenko VV, Vudmaska MI, Naidenov VG, Beletskii AV, Malisheva TA (2005). Patterns of expression of TSC-22 protein in astrocytic gliomas. Exp Oncol.

[19] Iida M, Anna CH, Gaskin ND, Walker NJ, Devereux TR (2007). The putative tumor suppressor Tsc-22 is downregulated early in chemically induced hepatocarcinogenesis and may be a suppressor of Gadd45b. Toxicol Sci.

[20] Yu J, Ershler M, Yu L, Wei M, Hackanson B, Yokohama A (2009). TSC-22 contributes to hematopoietic precursor cell proliferation and repopulation and is epigenetically silenced in large granular lymphocyte leukemia. Blood.

[21] Yoon CH, Rho SB, Kim ST, Kho S, Park J, Jang IS (2012). Crucial role of TSC-22 in preventing the proteasomal degradation of p53 in cervical cancer. PLoS One.

[22] Hashiguchi A, Okabayashi K, Asashima M (2004). Role of TSC-22 during early embryogenesis in Xenopus laevis. Dev Growth Differ.

[23] Jager J, Greiner V, Strzoda D, Seibert O, Niopek K, Sijmonsma TP (2014). Hepatic transforming growth factor-beta 1 stimulated clone-22 D1 controls systemic cholesterol metabolism. Mol Metab.

[24] Dohrmann CE, Noramly S, Raftery LA, Morgan BA (2002). Opposing effects on TSC-22 expression by BMP and receptor tyrosine kinase signals in the developing feather tract. Dev Dyn.

[25] Rysa J, Aro J, Ruskoaho H (2006). Early left ventricular gene expression profile in response to increase in blood pressure. Blood Press.

[26] Stanton LW, Garrard LJ, Damm D, Garrick BL, Lam A, Kapoun AM (2000). Altered patterns of gene expression in response to myocardial infarction. Circ Res.

[27] Prussak CE, Almazan MT, Tseng BY (1989). Peptide production from proteins separated by sodium dodecyl-sulfate polyacrylamide gel electrophoresis. Anal Biochem.

[28] Pfeffer MA, Pfeffer JM, Fishbein MC, Fletcher PJ, Spadaro J, Kloner RA (1979). Myocardial infarct size and ventricular function in rats. Circ Res.

[29] Tenhunen O, Soini Y, Ilves M, Rysa J, Tuukkanen J, Serpi R (2006). p38 Kinase rescues failing myocardium after myocardial infarction: evidence for angiogenic and anti-apoptotic mechanisms. FASEB J.

[30] Wang Y, Huang S, Sah VP, Ross J, Brown JH, Han J (1998). Cardiac muscle cell hypertrophy and apoptosis induced by distinct members of the p38 mitogen-activated protein kinase family. J Biol Chem.

[31] Niwa H, Yamamura K, Miyazaki J (1991). Efficient selection for high-expression transfectants with a novel eukaryotic vector. Gene.

[32] Tenhunen O, Rysa J, Ilves M, Soini Y, Ruskoaho H, Leskinen H (2006). Identification of cell cycle regulatory and inflammatory genes as predominant targets of p38 mitogen-activated protein kinase in the heart. Circ Res.

[33] Rysa J, Leskinen H, Ilves M, Ruskoaho H (2005). Distinct upregulation of extracellular matrix genes in transition from hypertrophy to hypertensive heart failure. Hypertension.

[34] Ronkainen VP, Ronkainen JJ, Hanninen SL, Leskinen H, Ruas JL, Pereira T (2007). Hypoxia inducible factor regulates the cardiac expression and secretion of apelin. FASEB J.

[35] Aro J, Tokola H, Ronkainen VP, Koivisto E, Tenhunen O, Ilves M (2013). Regulation of cardiac melusin gene expression by hypertrophic stimuli in the rat. Acta Physiol (Oxf).

[36] Koivisto E, Karkkola L, Majalahti T, Aro J, Tokola H, Kerkela R (2011). M-CAT element mediates mechanical stretch-activated transcription of B-type natriuretic peptide via ERK activation. Can J Physiol Pharmacol.

[37] Luosujarvi H, Aro J, Tokola H, Leskinen H, Tenhunen O, Skoumal R (2010). A novel p38 MAPK target dyxin is rapidly induced by mechanical load in the heart. Blood Press.

[38] Mustonen E, Leskinen H, Aro J, Luodonpää M, Vuolteenaho O, Ruskoaho H (2010). Metoprolol treatment lowers thrombospondin-4 expression in rats with myocardial infarction and left ventricular hypertrophy. Basic Clin Pharmacol Toxicol.

[39] Hoshijima M, Chien KR (2002). Mixed signals in heart failure: cancer rules. J Clin Invest.

[40] Brand T, Schneider MD (1995). The TGF beta superfamily in myocardium: ligands, receptors, transduction, and function. J Mol Cell Cardiol.

[41] Rosenkranz S, Flesch M, Amann K, Haeuseler C, Kilter H, Seeland U (2002). Alterations of beta-adrenergic signaling and cardiac hypertrophy in transgenic mice overexpressing TGF-beta(1). Am J Physiol Circ Physiol.

[42] Schultz Jel J, Witt SA, Glascock BJ, Nieman ML, Reiser PJ, Nix SL (2002). TGF-beta1 mediates the hypertrophic cardiomyocyte growth induced by angiotensin II. J Clin Invest.

[43] Schluter KD, Frischkopf K, Flesch M, Rosenkranz S, Taimor G, Piper HM (2000). Central role for ornithine decarboxylase in beta-adrenoceptor mediated hypertrophy. Cardiovasc Res.

[44] Schluter KD, Zhou XJ, Piper HM (1995). Induction of hypertrophic responsiveness to isoproterenol by TGF-beta in adult rat cardiomyocytes. Am J Physiol.

[45] Huntgeburth M, Tiemann K, Shahverdyan R, Schluter KD, Schreckenberg R, Gross ML (2011). Transforming growth factor beta(1) oppositely regulates the hypertrophic and contractile response to beta-adrenergic stimulation in the heart. PLoS One.

[46] Brouri F, Hanoun N, Mediani O, Saurini F, Hamon M, Vanhoutte PM (2004). Blockade of β1- and desensitization of β 2-adrenoceptors reduce isoprenaline-induced cardiac fibrosis. Eur J Pharmacol.

[47] De Carvalho FC, Sun Y, Weber KT (1997). Angiotensin II receptor blockade and myocardial fibrosis of the infarcted rat heart. J Lab Clin Med.

[48] Schieffer B, Wirger A, Meybrunn M, Seitz S, Holtz J, Riede UN (1994). Comparative effects of chronic angiotensin-converting enzyme inhibition and angiotensin II type 1 receptor blockade on cardiac remodeling after myocardial infarction in the rat. Circulation.

[49] Boluyt MO, Robinson KG, Meredith AL, Sen S, Lakatta EG, Crow MT (2005). Heart failure after long-term supravalvular aortic constriction in rats. Am J Hypertens.

[50] Kuoppala A, Shiota N, Lindstedt KA, Rysä J, Leskinen HK, Luodonpää M (2003). Expression of bradykinin receptors in the left ventricles of rats with pressure overload hypertrophy and heart failure. J Hypertens.

[51] Kato M, Wang L, Putta S, Wang M, Yuan H, Sun G (2010). Post-transcriptional up-regulation of Tsc-22 by Ybx1, a target of miR-216a, mediates TGF-{beta}-induced collagen expression in kidney cells. J Biol Chem.

[52] Jane-Lise S, Corda S, Chassagne C, Rappaport L (2000). The extracellular matrix and the cytoskeleton in heart hypertrophy and failure. Heart Fail Rev.

[53] Hino S, Kawamata H, Uchida D, Omotehara F, Miwa Y, Begum NM (2000). Nuclear translocation of TSC-22 (TGF-beta-stimulated clone-22) concomitant with apoptosis: TSC-22 as a putative transcriptional regulator. Biochem Biophys Res Commun.

